# Layer-specific quantification of myocardial deformation in sepsis-induced Takotsubo cardiomyopathy: Three case reports of a serial 2-dimensional speckle-tracking echocardiographic study

**DOI:** 10.1097/MD.0000000000005250

**Published:** 2016-11-04

**Authors:** Ming-Jui Hung, Yu-Cheng Kao, Wei-Siang Chen, Chun-Tai Mao, Tien-Hsing Chen, Ning-I. Yang, Ta Ko, Chung-Yu Liang

**Affiliations:** Section of Cardiology, Department of Medicine, Chang Gung Memorial Hospital, Keelung, Chang Gung University College of Medicine, Keelung City, Taiwan.

**Keywords:** cardiomyopathy, infection, sepsis, Takotsubo

## Abstract

**Introduction:**

Little is known about the time-course changes in left ventricular myocardial deformation in patients with Takotsubo cardiomyopathy (TC) using layer-specific quantification of myocardial deformation assessed by 2-dimensional speckle tracking echocardiography (2DSTE).

**Case summary:**

In this retrospective 2DSTE follow-up study of 3 female patients with sepsis-induced TC, we examined changes in strain among the 3 myocardial layers, and examined the changes in left ventricular diastolic function and right ventricular systolic function. In all 3 patients, there was improvement of at least 15% in left ventricular ejection fractions, and improvement in left ventricular longitudinal and circumferential strains. The absolute differences in left ventricular global strains between the endocardium and epicardium, and between the first and the third 2DSTE studies reflect the following: a decrease in all 3 myocardial layers in patients with acute TC; and a slower improvement in mid-myocardial and epicardial function during recovery of TC. In addition, the right ventricular free wall strains were also impaired in the acute stage of TC with gradual improvement during recovery.

**Conclusions:**

Left ventricular strains did not fully recover even 1 month after acute TC. In addition, right ventricular free wall strains were also impaired in all 3 patients initially. In this case series, we found that layer-specific 2DSTE is a more sensitive method for myocardial function assessment than standard echocardiography.

## 1 Introduction

Takotsubo cardiomyopathy (TC) is a transient cardiac syndrome characterized by reversible left ventricular dysfunction, with apical ballooning and electrocardiographic changes mimicking acute coronary syndrome without obstructive coronary artery disease.^[1–3]^ TC most often occurs in postmenopausal women and is usually triggered by physical or psychological stress, although neurological disturbances have also been reported as triggers.^[4–7]^ Abnormal systolic and diastolic mechanics exist in the acute stage of TC.^[5]^ A recent cardiac magnetic resonance imaging study found that systolic function recovered faster than diastolic function in patients with TC.^[8]^ Unlike the right ventricle, the left ventricle is composed of 3 myocardial layers, namely endocardial, mid-myocardial, and epicardial myocardium. Little is known about the time-course changes in left ventricular myocardial deformation in patients with TC using layer-specific quantification of myocardial deformation assessed by 2-dimensional speckle tracking echocardiography (2DSTE). In this retrospective 2DSTE follow-up study of patients with sepsis-induced TC, we examined changes in strain among the 3 myocardial layers and examined the changes in left ventricular diastolic function and right ventricular systolic function.

## 2 Clinical findings, diagnostic methods, therapeutic methods, therapeutic interventions, and follow-up echocardiograms

During the period July 2014 to June 2015, in all, 3 consecutive patients were admitted to our intensive care unit because of sepsis and clinically acute congestive heart failure (New York Heart Association functional class IV). Since acute infectious diseases are a contraindication for coronary angiography, none of the patients underwent coronary angiography to define coronary artery disease, but instead were treated for the underlying acute urinary tract infection. Echocardiographic examinations in all patients revealed a left ventricle with a “lobster trap” appearance characteristic of TC. Therefore, the preliminary diagnosis in all 3 patients was urinary tract infection-induced TC. The patients underwent 3 serial 2DSTE studies: the first on admission, the second 7 to 14 days after admission, and the third before discharge (case 1) or 1 to 2 months after admission (cases 2 and 3). The follow-up protocols were designed to confirm the diagnosis of TC. Clinical data definitions were the same as those reported in our previous study.^[9]^

All echocardiographic examinations were performed using a commercially available system (Vivid E9, General Electric-Vingmed, Milwaukee, WI). Standard echocardiographic and 2DSTE techniques were performed as reported in our previous study.^[10]^ Analyses of layer-specific longitudinal and circumferential strains were obtained from 3 apical views and 3 parasternal short-axis views.^[11]^ The data on systolic longitudinal and circumferential strains from endocardial, mid-myocardial, and epicardial myocardium were obtained using off-line software (General Electric-Vingmed, Milwaukee, WI) in 17 longitudinal and 6 circumferential segments. All segmental values were averaged to achieve global longitudinal and circumferential strains for each myocardial layer, respectively. The present study was approved by institutional review board of Chang Gung Memorial Hospital (No. 104–9129B). Herein, we present the results of the follow-up serial electrocardiographic and 2DSTE studies in the 3 patients with sepsis-induced TC.

### 2.1 Case 1

**Figure 1:**
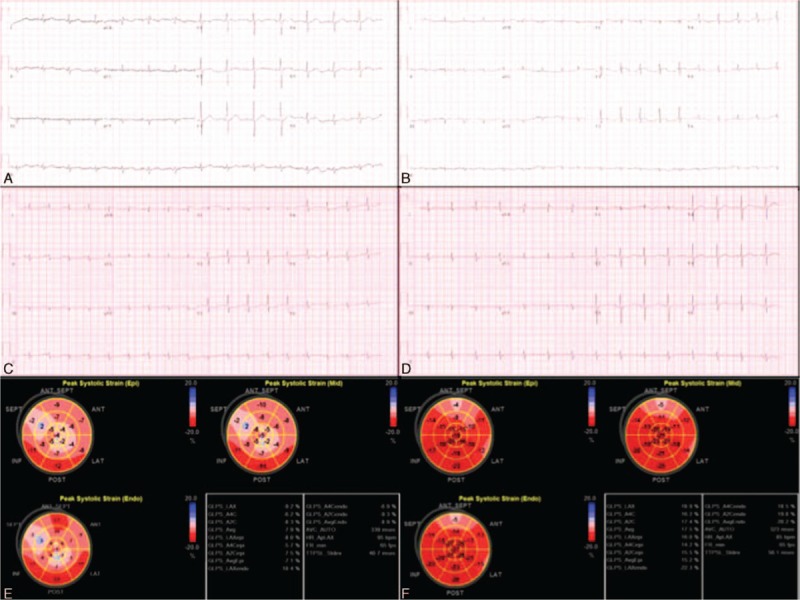
Electrocardiograms and image studies in case 1. Serial electrocardiograms show normal electrocardiogram 2 months before admission (A), ST-elevation in leads V_2-3_ on the second day (B), ST-depression and T-wave inversion in leads V_3-5_ on the fourth day (C), and nearly normal on the 39th day after admission (D). Three-layer left ventricular global longitudinal strain studies show diffusely impaired systolic strains on the first (E), and improvement on the 12th day (F).

An 82-year-old woman presented to the emergency department with a 1-day history of rapidly progressive shortness of breath. Her underlying comorbidities included type II diabetes mellitus and chronic hepatitis B and C virus infection. The patient's medical history included cervical cancer that had been successfully treated with radiotherapy. Blood hemograms and biochemical test results were indicative of sepsis. Subsequent urine culture yielded *Escherichia coli*. The 12-lead electrocardiogram on the second day after admission showed ST-segment elevation in precordial leads with an initial troponin-I level of 1.622 ng/mL. The patient presented with dyspnea at rest. Transthoracic echocardiography revealed characteristics of TC and a left ventricular ejection fraction of 26%. The findings on follow-up electrocardiographic and 2DSTE studies were consistent with a diagnosis of sepsis-induced TC (Fig. 1).

### 2.2 Case 2

**Figure 2:**
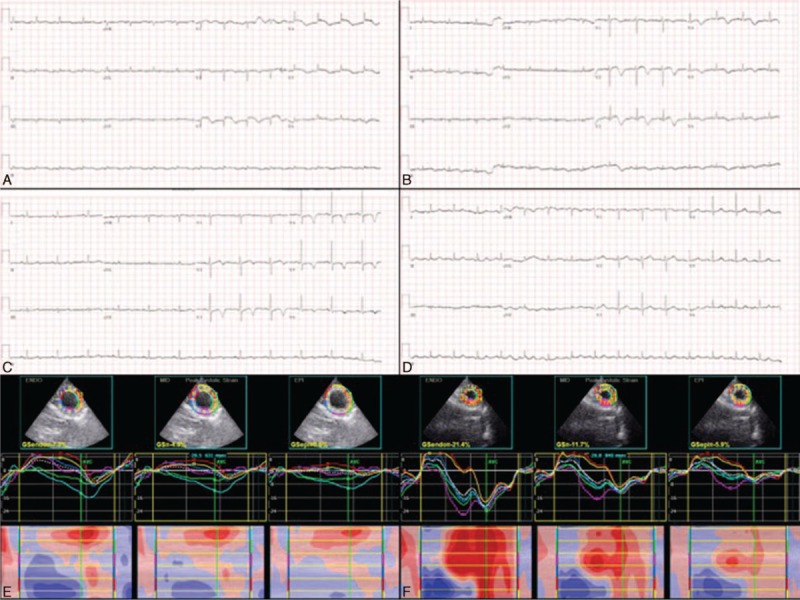
Electrocardiograms and 2-dimensional speckle tracking images in case 2. Diffuse ST-elevation in precordial leads on the 14th day (A), T-wave inversions in the same leads on the 26th day (B) and 75th day (C), and normalized ST-T changes 8 months after admission (D). Three-layer left ventricular global circumferential strain studies show transmurally impaired systolic strains on the 14th day (E) and improvement on the 55th day (F).

An 80-year-old woman was transferred from the ordinary ward to the intensive care unit because of a 2-day history of intermittent fever and chills. Her underlying comorbidities included type II diabetes mellitus, hypertension, and stage V chronic kidney disease. Blood culture grew *Klebsiella pnmeumoniae*. Her 12-lead electrocardiogram and transthoracic echocardiogram obtained 2 weeks after admission demonstrated characteristics of TC. The initial troponin-I and B-type natriuretic peptide levels were 6.116 ng/mL and 1160 pg/mL, respectively. The findings on follow-up electrocardiographic and 2DSTE studies were consistent with a diagnosis of sepsis-induced TC (Fig. 2).

### 2.3 Case 3

**Figure 3:**
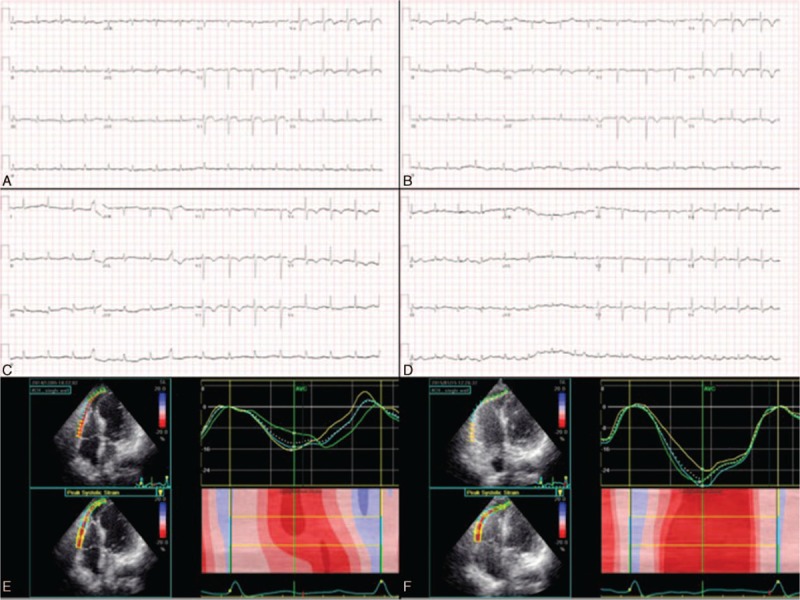
Electrocardiograms and right ventricular free wall strains in case 3. ST-elevation in V_3-5_ leads on the fourth day (A), T-wave inversions in the same leads on the 14th day (B) and 44th day (C), and normalized ST-T changes 11 months after admission (D). Reduced right ventricular free wall strains initially (E), which were relieved 1.5 months later (F).

A 68-year-old woman presented to the emergency department with a 1-day history of intermittent fever and chills. Underlying comorbidities included chronic hepatitis C, type II diabetes mellitus, and stage IV chronic kidney disease. Her medical history included liver cirrhosis Child-Pugh B and right partial lobectomy for hepatocellular carcinoma. Urine and blood cultures grew *E coli*. Her initial 12-lead electrocardiogram and transthoracic echocardiogram were suggestive of TC. The initial troponin-I level was 0.767 ng/mL. The findings on follow-up electrocardiographic and 2DSTE studies were consistent with a diagnosis of sepsis-induced TC (Fig. 3).

All 3 patients in this case series were older and were admitted to our hospital because of sepsis complicated by TC. The patients were discharged uneventfully after intravenous antibiotic treatment. Table 1 shows the serial data from the standard echocardiographic and 2DSTE studies. In all 3 patients, there was improvement of at least 15% in left ventricular ejection fractions, and improvement in left ventricular longitudinal and circumferential strains. The absolute differences in left ventricular global strains between the endocardium and epicardium, and between the first and the third 2DSTE studies reflect the following: a decrease in all 3 myocardial layers in patients with acute TC; and a slower improvement in mid-myocardial and epicardial function during recovery of TC. In addition, the right ventricular free wall strains were also impaired in the acute stage of TC with gradual improvement during recovery. Interestingly, we also found that the left ventricular isovolumic relaxation time indexed by heart rate—a parameter of left ventricular diastolic function—was transiently prolonged during recovery of TC.

**Table 1:**
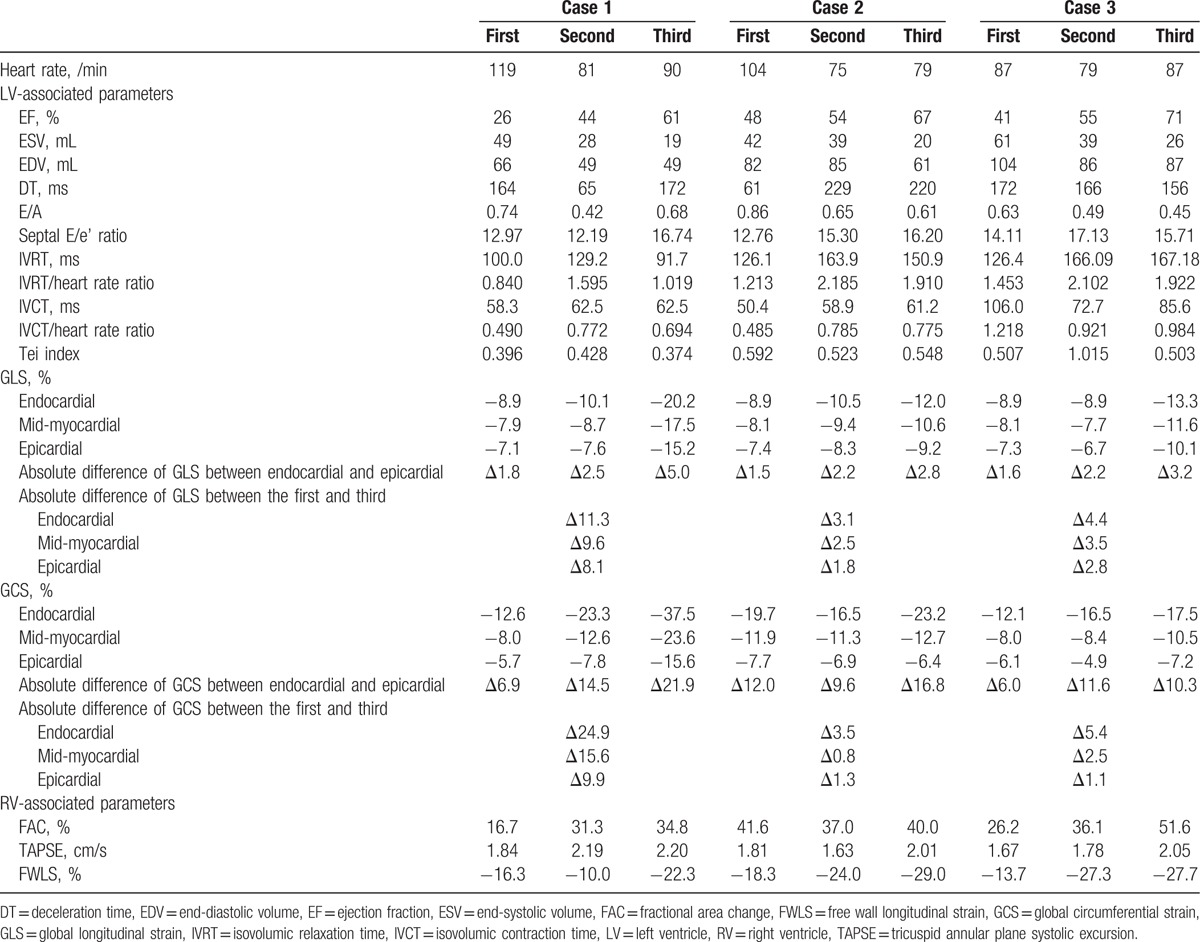
Serial echocardiographic data of 3 patients with Takotsubo cardiomyopathy.

## 3 Discussion

We have demonstrated that left and right ventricular systolic function, and left ventricular diastolic function are impaired by sepsis-induced TC. Although left ventricular ejection fractions had normalized at the last echocardiographic follow-up, the left ventricular strains were not similar to those in normal subjects.^[11,12]^ This explains at least in part why ejection fraction alone is not a sensitive parameter for assessing systolic function. We found that the global left ventricular longitudinal strains in all 3 patients and the circumferential strains in patients 2 and 3 had not returned to normal even 1 month after admission. The endocardium undergoes greater dimensional changes during systole than does the epicardium in healthy myocardium.^[13]^ The contraction of muscle fiber in the mid-wall, which is linearly related to circumferential strain,^[14]^ better reflects intrinsic contractility than contraction of fibers in the endocardium. Consistent with these findings, our data show that the circumferential strain improved slower than the longitudinal strain, especially in the mid-myocardial and epicardial layers. This indicates that the global left ventricular systolic function had not completely normalized even after the left ventricular ejection fraction had normalized. The ejection fraction is geometric changes of ventricle rather than contractile function measurements of myocardium. Therefore, 2DSTE provides more information for assessing cardiac muscular function than standard echocardiography.

A recent investigation found that sepsis-triggered TC may be the cause of sepsis-induced myocardial depression, and that left ventricular dysfunction is reversible within 1 to 2 weeks.^[15]^ The authors also found that the right ventricle was involved in a quarter of the cases of sepsis-induced TC. In the present study, the right ventricle was involved in all 3 cases of sepsis-induced TC, and the improvement in right ventricular free wall strain was associated with improvement in left ventricular ejection fraction and strain. We also noted that the strain was lowest in the apex of the right ventricular free wall (in absolute level). Contrary to left ventricular global strains, the global longitudinal right ventricular free wall strains in the 3 patients all recovered to normal (<−20%, ie, >20% in absolute level).^[16]^ The thinner myocardial width of the right ventricle as compared with that of the left ventricle might be partly responsible for this finding. Studies have demonstrated that right ventricular free wall strain analysis provides prognostic information in patients with cardiovascular disease.^[17–19]^ The mechanisms underlying right ventricular involvement in acute TC remain elusive. Some investigators suggest that intensive contraction of the base can introduce increased preload wall stress on the apical myocardial segments, thus causing apical akinesis. Therefore, TC and McConnell sign may share a common mechanism in the development of right ventricular apical akinesis.^[20,21]^

Ahtarovski et al^[8]^ recently reported that diastolic recovery is slower than systolic recovery in patients with TC. They hypothesized that apical edema and calcium mishandling might contribute to the persistent diastolic dysfunction seen in patients with TC. In the present case series, we found that left ventricular diastolic function recovered as the left ventricular ejection fraction recovered as reflected by the transiently prolonged left ventricular isovolumic relaxation time.

## 4 Conclusions

Left ventricular strains, one of the systolic function parameters, did not fully recover even 1 month after acute TC. In addition, right ventricular free wall strains were also impaired in all 3 patients initially. In this case series, we found that layer-specific 2DSTE is a more sensitive method for myocardial function assessment than standard echocardiography. Based on our findings, we suggest that follow-up 2DSTE studies be performed in patients with TC even after left ventricular ejection fraction has normalized.
